# ALS as a distal axonopathy: molecular mechanisms affecting neuromuscular junction stability in the presymptomatic stages of the disease

**DOI:** 10.3389/fnins.2014.00252

**Published:** 2014-08-14

**Authors:** Elizabeth B. Moloney, Fred de Winter, Joost Verhaagen

**Affiliations:** ^1^Department of Regeneration of Sensorimotor Systems, Netherlands Institute for Neuroscience, Institute of the Royal Netherlands Academy of Arts and ScienceAmsterdam, Netherlands; ^2^Department of Neurosurgery, Leiden University Medical CentreLeiden, Netherlands; ^3^Centre for Neurogenomics and Cognitive Research, Vrije Universiteit AmsterdamAmsterdam, Netherlands

**Keywords:** Amyotrophic Lateral Sclerosis, distal axonopathy, axon guidance molecules, neuromuscular junction, motor neuron, skeletal muscle, terminal Schwann cell

## Abstract

Amyotrophic Lateral Sclerosis (ALS) is being redefined as a distal axonopathy, in that many molecular changes influencing motor neuron degeneration occur at the neuromuscular junction (NMJ) at very early stages of the disease prior to symptom onset. A huge variety of genetic and environmental causes have been associated with ALS, and interestingly, although the cause of the disease can differ, both sporadic and familial forms of ALS show a remarkable similarity in terms of disease progression and clinical manifestation. The NMJ is a highly specialized synapse, allowing for controlled signaling between muscle and nerve necessary for skeletal muscle function. In this review we will evaluate the clinical, animal experimental and cellular/molecular evidence that supports the idea of ALS as a distal axonopathy. We will discuss the early molecular mechanisms that occur at the NMJ, which alter the functional abilities of the NMJ. Specifically, we focus on the role of axon guidance molecules on the stability of the cytoskeleton and how these molecules may directly influence the cells of the NMJ in a way that may initiate or facilitate the dismantling of the neuromuscular synapse in the presymptomatic stages of ALS.

## Introduction

Amyotrophic Lateral Sclerosis (ALS), first described in the literature 150 years ago by French neurologist Jean-Martin Charcot, is a highly debilitating disease caused by progressive degeneration of upper and lower motor neurons. Denervated muscles weaken and atrophy, and death usually occurs due to respiratory failure. ALS is an aggressive disease, with many patients dying within 3–5 years of diagnosis. Ninety percent of cases are considered sporadic (sALS), with varied environmental and/or genetic factors influencing the pathophysiology. The discovery of a direct genetic cause for ALS came in the early 1990s in the form of mutations in the superoxide dismutase-1 gene (SOD1; Rosen et al., 1993), which account for approximately 20% of familial ALS (fALS) cases. To date, over 150 different mutations in SOD1 have been linked with the disease, some causing a long clinical course (e.g., H46R) while others trigger an exceptionally aggressive form of the disease (e.g., A4V; Redler and Dokholyan, 2012). Mutations in other genes (alsin, senataxin, angiogenin, profilin, VAMP-associated protein B, dynactin, TAR DNA-binding protein-43 [TDP-43] or fused in sarcoma [FUS]) account for another 10% of fALS cases (Ludolph et al., 2012; Robberecht and Philips, 2013; Renton et al., 2014). A large portion of the remaining fALS cases (~50%) have now been attributed to an expansion of the intronic hexanucleotide repeat sequence in C9ORF72 (Renton et al., 2011).

It is becoming clearer that genetic factors may still play a role in apparently sporadic disorders and this is illustrated by the discovery of an increasing number of single nucleotide polymorphisms (SNPs) linked to sALS across various populations and ethnicities across the world (Laaksovirta et al., 2010; Shatunov et al., 2010; Fogh et al., 2014). Various SNPs in chromosome 9 (which also contains the C9ORF72 sequence connected to the majority of fALS cases, see above) associate with the haplotype for frontotemporal dementia, supporting the idea of shared genetic causes for ALS and other neurodegenerative disorders (Laaksovirta et al., 2010; Shatunov et al., 2010). Overlap between particular pathways linked to ALS and Parkinson's disease pathophysiology had previously been identified by Lesnick and colleagues, who found that SNPs in certain genes within the axon guidance pathways in PD associated with ALS susceptibility, survival and age of disease onset (Lesnick et al., 2008). Recently SNPs in chromosome 17 were identified via the largest association study to date based on over 13,000 individuals from around the world, the lead SNPs being localized in a gene related to Wallerian degeneration, a mechanism that shares morphological features to the axonal dying back process in ALS (Fogh et al., 2014).

In terms of the environmental aspects linked to sporadic ALS pathophysiology an interesting cluster of sALS has been found among (retired) American football or Italian soccer players (Chiò et al., 2005, 2009; Lehman et al., 2012), and is thought not to be due to physical exercise *per se*, but rather a result of head injury inherent to the sport itself. Ingestion of cyanobacteria-contaminated bat meat or cycad seed derivatives was linked with an ALS-like phenotype in inhabitants of the island of Guam and was eventually shown to be caused by neurotoxins produced by the cyanobacteria (Tabata et al., 2008; Bradley et al., 2013; De Munck et al., 2013). A comparable clustering of sALS cases in Gulf war veterans was attributed to the inhalation of desert dust containing traces of cyanobacteria (Cox et al., 2009). Prior viral infections (e.g., HIV) have also been linked to an increased risk of an already susceptible individual to developing a neurodegenerative disease, including ALS (Zhou et al., 2012; Alfahad and Nath, 2013). Other environmental causes, such as exposure to heavy metals, pesticides, or lifestyle differences have been put forward, but require further analysis to convincingly support a causal link to sALS (Weisskopf et al., 2009; Trojsi et al., 2013).

Due to the wide variety of genes and mutations now identified for fALS and the discovery of other (*de novo)* genetic and/or varied environmental factors influencing sALS pathogenesis, it has become clear that ALS has a highly varied etiology, which ultimately converges to produce similar clinical symptoms. Care is currently based on symptomatic treatment as there is no effective disease-modifying therapy (reviewed in Phukan and Hardiman, 2009). The only approved medical treatment is Riluzole, a presynaptic glutamate release inhibitor, which merely extends life by 3–5 months (Miller et al., 2012). Nutritional management with the aid of parenteral tubing can prevent excessive weight loss and dehydration and may improve quality of life, but has marginal survival benefits (Forbes et al., 2004). However, respiratory management by non-invasive ventilation has improved survival, albeit in a subset of patients (Bourke et al., 2006).

In this review we will evaluate the clinical, animal experimental and cellular/molecular evidence that supports the idea of ALS as a distal axonopathy, which proposes that pathological changes occur at the neuromuscular junction (NMJ) prior to motor neuron degeneration and onset of clinical symptoms. In addition, we will discuss the early molecular mechanisms occurring at the NMJ that may be inducing a loss in synaptic integrity by altering the stability of the distal cytoskeleton in the presymptomatic phase of the disease, initiating a cascade of events that instigate the distal axonopathy phenotype. We will specifically focus on the role of axon guidance molecules in dismantling the NMJ, emphasizing on their capacity to influence the stability of the distal cytoskeleton of the motor neuron during the presymptomatic stages of ALS.

## Early clinical manifestations of ALS

Clinical assessment of neuromuscular performance in ALS patients has been the focus of many studies over the last decades (Dengler et al., 1990; Killian et al., 1994; Schmied et al., 1999; Fischer et al., 2004; Noto et al., 2011). Electrophysiological changes are one of the first measurable alterations to occur and form a large part of the tests used to diagnose ALS in humans; the (modified) El Escorial criteria (De Carvalho et al., 2008) alongside the Awaji criteria (Okita et al., 2011) provide electrophysiological data, and this, combined with the ALS functional-rating scale (Cedarbaum and Stambler, 1997) provides a more definitive diagnosis. Nerve conduction measurements such as Muscle Fiber Conduction Velocity (MFCV) can be 89% effective at predicting the development of ALS in patients who already show denervation in muscle biopsies but do not yet conform to the other ALS diagnostic tests mentioned above (Blijham et al., 2007). Altered axon excitability in ALS patients has been observed to occur in a distal to proximal fashion (Kanai et al., 2006; Nakata et al., 2006; Vucic and Kiernan, 2006). *In vivo* data from animal studies indicates an increase in electrical excitability of hypoglossal motor neurons can occur as early as 4 days after birth in the G93A-hSOD1 ALS mouse (Van Zundert et al., 2008). Changes in fasciculation potentials has been recently shown to precede and anticipate NMJ instability and reinnervation, and is consistent with a very early phase of increased axonal excitability (de Carvalho and Swash, 2013). Axonal alterations are not only confined to motor neurons as clinical evidence also suggests that large-caliber myelinated fibers undergo axonal loss to produce sensory abnormalities in some patients (Hammad et al., 2007).

## Theories on the cause(s) of sALS

Over the last decades, many studies have identified potential causes for sALS, some of which include excitotoxicity, astroglia, and/or microglia dysfunction, oxidative stress and mitochondrial dysfunction, endoplasmic reticulum stress, defects in RNA processing, growth factor abnormalities, defects in axonal transport, metabolic alterations and accumulation of protein aggregates (reviewed in Robberecht and Philips, 2013). However, no research has pointed toward a common pathway to collate these various hypotheses, making it almost impossible to create an effective neuroprotective therapy, as individual treatments for each of these potential causes inevitably “ignores” the other factors involved in the disease. Combinatorial treatments are a potential solution, yet, the question still remains which causes do you target if there is no direct knowledge about when these different biological dysfunctions occur over the course of the disease? Ideally, a therapy should target the initial and early changes common to all forms of ALS. Thus, the quest continues to unify the early biological causes of ALS and determine the key factors that initiate the disease.

Although ALS has traditionally been described as a disease of the motor neuron cell body, several studies using mutant SOD1 mice have illustrated the non-cell-autonomous pathogenic nature of the disease. For a comprehensive review on glial-neuronal interactions contributing to ALS, see Valori et al. (2014). The well-studied G93A-hSOD1 ALS mouse, which models a subset of the inherited disease based on mutations in the superoxide dismutase-1 gene (SOD1), has been a vital tool in progressing our understanding of ALS; this mouse exhibits behavioral symptom onset at postnatal day 90 (P90) and death by P120-140 caused by a gain-of-function toxicity mediated by the mutant SOD1 enzyme (Gurney et al., 1994). Mutant SOD1-related damage (i.e., oxidative stress) in motor neurons is necessary in determining the onset of disease initiation, whereas limiting G93A-hSOD1 expression to microglia and/or astrocytes accelerates disease progression without affecting the onset (Boillée et al., 2006; Yamanaka et al., 2008a,b). On the other hand, accumulation of or limiting the expression of a dismutase active mutant SOD1 to Schwann cells delays disease onset (Lobsiger et al., 2009; Turner et al., 2010). These studies demonstrate that various cells can have differing functions (destructive or supportive) in terms of certain molecular features of the disease but work in parallel to produce the typical ALS pathophysiology. Another study focused on the presymptomatic changes in non-neuronal cells and showed that an increase in GFAP expression, indicative of activated astrocytes, is detected in the lumber spinal cord of ALS mice as early as 25 days of age (Keller et al., 2009). With this in mind, it is possible that early alterations in the astrocyte-motor neuron relationship create a vulnerable environment for the neuronal cell body which may ultimately affect the survival of the motor neuron (reviewed in Rafalowska et al., 2010; Phatnani et al., 2013). Degenerative changes may also be occurring in oligodendrocytes: a loss of oligodendrocytes in both ALS patients and presymptomatic ALS mice has been described, and newly differentiated oligodendrocytes that form to compensate for this loss have a reduced expression of monocarboxylate transporter 1 (MCT-1) rendering them dysfunctional in terms of the metabolic support they provide for motor neurons (Lee et al., 2012; Philips et al., 2013). MCT-1 is expressed in neurons and other glial cells, but it is the oligodendrocyte derived MCT-1 that provides metabolic support for axons as demonstrated by the axon degeneration and subsequent motor neuron loss that occurs after oligodendrocyte-specific knockdown of MCT-1 (Lee et al., 2012). Thus, oligodendrocytes, too, play a role in motor neuron survival and as such are important players in ALS-related motor neuron degeneration.

In recent years, the “dying-back” hypothesis has obtained much attention in the context of ALS pathophysiology. According to this hypothesis, motor neurons and nerve terminals show pathological changes prior to motor neuron degeneration and the onset of clinical symptoms. Specifically, it proposes that ALS is a distal axonopathy, whereby changes first occur distally at the NMJ itself and progress proximally toward the cell body. Interestingly, evidence of distal axonopathy has been found in other neurodegenerative diseases like Alzheimer's disease or Parkinson's disease, where axonal defects occur prior to cell death and the loss of axonal function correlates strongly with the onset of functional decline (Selkoe, 2002; Dauer and Przedborski, 2003; Arendt, 2009).

Using mutant SOD1 ALS mouse models, several seminal papers have provided experimental evidence for ALS as a distal axonopathy. Neuromuscular synapses differ in terms of their anatomical plasticity and vulnerability to denervation, with the most vulnerable (i.e., fast-fatigable; FF) synapses being lost early in the disease process (Frey et al., 2000). Fischer and colleagues observed that denervation of FF synapses at Type IIb/x muscle fibers (at 47 days of age) and ventral root axon loss (at 80 days of age) was occurring prior to loss of the cell body (at 100 days of age) in the G93A-hSOD1 mouse (Fischer et al., 2004). Fischer and colleagues also provided the first histological report of the dying-back phenomenon occurring in humans in the form of a patient with sALS who died unexpectedly: denervation and innervation changes at the muscle were observed whereas pathological changes in the motor neuron itself were not detected (Fischer et al., 2004). This extends the electrophysiological observations of Dengler and colleagues which were indicative of very early changes in the function of distal peripheral nerves in patients presenting with early muscle weakness (Dengler et al., 1990). More recent evidence shows that presymptomatic changes in electrophysiological parameters are predictive of clinical onset and survival in the ALS mouse (Mancuso et al., 2011, 2014; Casas et al., 2013). In addition, neuromuscular denervation appears to occur independently to the activation of the cell death pathway in motor neurons: ALS mice in which motor neuron death is completely abolished still develop the disease although there is a modest increase in survival due to a delay in muscle denervation and symptom onset (Kostic et al., 1997; Gould et al., 2006). These studies support the idea that neuromuscular denervation and symptom manifestation in ALS can occur regardless of motor neuron survival, implicating that alterations elsewhere, e.g., in skeletal muscle, muscle satellite cells or terminal Schwann cells (TSCs) are able to influence axonal integrity, thus challenging the “neurocentric” view of ALS (reviewed in Pansarasa et al., 2014).

Anatomically speaking, motor neurons synapsing on skeletal muscles can be categorized into several functional classes: fast-fatigable (FF), fast-fatigue resistant (FR) and slow (S). Skeletal muscles are innervated by a characteristic composition of FF (synapsing on Type IIb/x muscle fibers), FR (synapsing on Type IIa muscle fibers) and S (synapsing on Type I muscle fibers) motor units, which provide the distinct force and twitch features that define the functional properties of the individual muscle (Burke et al., 1973). A motor unit is characterized by one motor neuron and all the skeletal muscle fibers innervated by that axon. FF motor units on Type IIb/x fibers are the most vulnerable and the earliest to be lost in both human and rodent ALS (Dengler et al., 1990; Frey et al., 2000; Atkin et al., 2005; Pun et al., 2006; Saxena et al., 2009). Over the course of the disease, the preferential denervation of Type IIb/x muscle fibers progressively leads to an increase of Type I and IIa muscle fibers due to activity-dependent conversion of the muscle fibers and collateral sprouting of surviving motor units (Hegedus et al., 2007, 2008; Gordon et al., 2010). Fibers that remain denervated atrophy, and it is this constant ebb-and-flow of denervation and reinnervation by reduced-force capacity motor neurons that gives ALS its characteristic feature of muscle weakness.

Interestingly, the selective vulnerability of FF synapses also occurs in aged or induced-paralysis paradigms and coincides with a lack of anatomical plasticity (Frey et al., 2000; Valdez et al., 2012). On the other hand, slow type synapses, which are more resistant to ALS and are the last to be affected, are capable of stimulus-induced sprouting (Frey et al., 2000). This is consistent with the idea that individual motor pools contain two distinct types of motor neurons: those which degenerate and those which can reinnervate (Schaefer et al., 2005), indicative of motor neuron-intrinsic factors playing a role in this apparent selective vulnerability. For example, preferential denervation of FF motor units occurs in mice lacking wild-type SOD1 illustrating that these synapses are especially vulnerable to oxidative injury (Fischer et al., 2011, 2012). Vulnerable motor neurons are also more prone to endoplasmic reticulum stress, with an unfolded protein response occurring at least 4 weeks prior to the earliest denervation (Saxena et al., 2009). The extra-ocular muscle remains largely innervated even in the terminal stages of the disease (Tjust et al., 2012), and a recent study using end-stage ALS patient material found significant decreases in synaptic protein levels at the NMJs of limb muscles compared to extra-ocular muscles that could explain this phenomenon (Liu et al., 2014). In addition, motor neurons that are resistant to degeneration express higher levels of calcium buffering proteins which may allow them to be more resistant against excitotoxic stimuli and may explain the resistance of the oculomotor and Onuf's nucleus to degeneration in ALS (Obál et al., 2006). Interestingly, pharmacological modulation of calcium at the NMJ using calpain inhibitors has been shown to protect the distal cytoskeleton from degradation in cases of autoimmune peripheral neuropathies (O'Hanlon et al., 2003), and support the idea of protecting the distal cytoskeleton as a therapeutic approach for preventing or delaying distal axonopathies (see below for more discussion on the role of the axonal cytoskeleton and ALS pathogenesis).

Mitochondrial (dys)function has also been linked to several severe neurodegenerative diseases, ALS included (Filosto et al., 2011; Narendra and Youle, 2012). In spinal motor neurons, misfolded mutant SOD1 protein preferentially binds to mitochondrial membranes (Vande Velde et al., 2008) and accumulation of SOD1 mutations in mitochondria leads to organelle dysfunction, oxidative stress and subsequent defects in neuronal physiology (Liu et al., 2004; Deng et al., 2006). Interestingly, clustering of mitochondria and mutant SOD1 in G93A-hSOD1 rats into discrete domains within the motor neuron creates dysfunctional regions along the axon which can lead to progressive axonopathy (Sotelo-Silveira et al., 2009). In addition, overexpression of mutant SOD1 in the intermembrane space of mitochondria results in phenotypic features similar to ALS such as weight loss, muscle weakness and motor impairment, but with no evidence of muscle denervation (Igoudjil et al., 2011). Defects in mitochondrial DNA in human skeletal muscle have recently been observed in both sporadic and familial forms of ALS (Artuso et al., 2013) illustrating that it is not only dysfunction in motor neuron mitochondria that can affect the disease process. Enhancing the calcium buffering capacity of mitochondria in mutant SOD1 ALS mice suppressed motor neuron death over the course of the disease, but did not ameliorate muscle denervation, motor axon degeneration, disease progression or survival (Parone et al., 2013). Recently, abnormal mitochondrial dynamics in skeletal muscle of presymptomatic G93A-hSOD1 mice was discovered to directly relate to muscle dysfunction (a feature which occurs early in disease progression) and therefore may be actively promoting ALS progression (Luo et al., 2013). Thus, given that mitochondrial dysfunction appears in many neurodegenerative diseases and is not restricted to the vulnerable subpopulation of cells affected by the disease, it is unlikely that they are primary contributors in initiating the disease process; instead, defects in mitochondrial physiology may play a role in amplifying the pathogenic process (reviewed in Dupuis, 2014).

Defective RNA processing is emerging as a common feature of many neurodegenerative diseases (Belzil et al., 2013). Several lines of evidence illustrate how aberrant RNA processing due to changes in TDP-43, FUS, and/or C9ORF72 may directly play a role in the pathogenesis of both fALS and sALS (Droppelmann et al., 2014; Raman et al., 2014). Mutations in the TDP-43, FUS and C9ORF72 genes have been shown to alter normal RNA processing which may, in turn, affect important protein homeostasis causing (detrimental) changes in neuronal function (Polymenidou et al., 2012; Ling et al., 2013). TDP-43 can be actively transported within motor neurons to regulate axonal growth by binding to and therefore modulating the fate of axonal mRNA in the distal regions of the neuron (Fallini et al., 2012). TDP-43 itself also plays a role in the transport of stress granules (vesicles containing mRNA transcripts) to the distal axon, with ALS-linked mutations in TDP-43 causing impairment of this transport (Alami et al., 2014). Interestingly, mutant TDP-43 results in altered RNA splicing behavior of TDP-43 targets such as genes involved in synaptic transmission and function already in the early stages of the disease (Arnold et al., 2013). In addition, mislocalization, or misregulation of mRNA transcripts to or at the distal compartment may itself impair synaptic function due to a deficiency of important components at the nerve terminal (Mikl et al., 2010). FUS and TDP-43 share similar roles in terms of RNA processing, thus many of the effects mentioned above for TDP-43 can be mediated by FUS also (Lagier-Tourenne et al., 2012; Ling et al., 2013; Deng et al., 2014). Of specific importance to the distal axonopathy hypothesis of ALS, FUS has been linked with modulating synaptic transmission at the NMJ by affecting the quantity of neurotransmitter release (Machamer et al., 2014), which can directly impact synaptic function.

Given that it has only recently been discovered as a genetic cause for ALS, there is still much work to be done to decipher the mechanism through which mutant C9ORF72 contributes to ALS (Renton et al., 2011; Liu et al., 2014). A recent review highlights the possible mechanisms of action (Gendron et al., 2014), but of most interest here is the idea that mutant C9ORF72 protein may sequester other RNA/DNA-binding proteins, such as TDP-43, preventing their normal function, and thus may influence neuronal function indirectly by the processes regulated by TDP-43 as described above.

TDP-43, FUS and C9ORF72 have also been linked with the formation of insoluble, cytoplasmic aggregates characteristic of degenerating motor neurons in ALS (Blokhuis et al., 2013) and although the impact of protein aggregates in disease pathology is still being determined, one could see these inclusions as physical barriers preventing transport of vital cargos (mRNA transcripts or protein) to the distal axon, and thus interfere with synaptic function which in turn affects motor neuron health. In addition, mutant TDP-43 and FUS proteins may also contribute to ALS pathology by affecting the biogenesis of microRNAs, which are known to have an important role in motor neuron and NMJ function (Williams et al., 2009; Kye and Gonçalves Ido, 2014).

Thus, defective RNA processing as mediated by changes in TDP-43, FUS or C9ORF72 may influence synaptic function as a trigger for motor neuron degeneration. Impairment of synaptic function as an early pathological feature in the distal axonopathy hypothesis of ALS will be further discussed below.

## Distinctive axonal and/or synaptic features that could initiate a motor neuron disease phenotype

**Figure 1:**
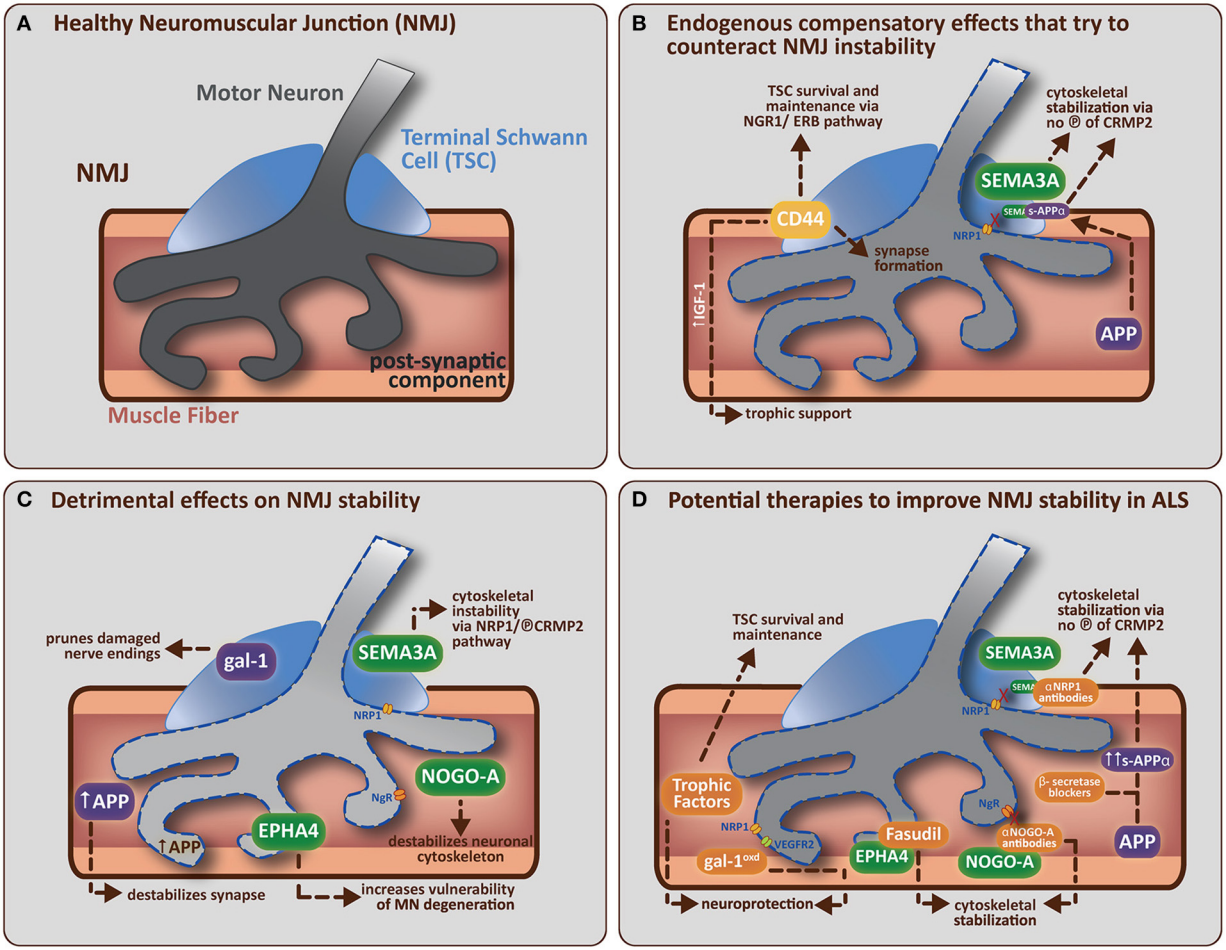
**(A)** A healthy neuromuscular junction (NMJ) is a tripartite synapse composed of a motor neuron (MN) terminal synapsing onto a muscle fiber. Terminal Schwann cells (TSCs) that envelop the junction have a role in supporting and maintaining the motor neuron terminal. **(B)** Potential endogenous compensatory effects that could counteract NMJ instability in ALS. CD44 is upregulated by TSCs in ALS mice and is known to encourage IGF-1 production to provide trophic support to damaged muscle fibers. CD44 has also been found to colocalize with ErbB, and can amplify the NRG1-ErbB pathway with two potential outcomes (1) indirectly via paracrine stimulation of the NRG1-ErbB pathway in MN, which is involved in synapse formation/maturation and/or (2) directly via NRG1-ErbB pathway in TSCs, which is involved in survival and maintenance of TSC (vital for correct synapse function). APP (in muscle) can be cleaved (by α-secretase followed by γ-secretase) to form secreted-APP-α (sAPP-α), which can bind CRMP2 (and maintain it in a non-phosphorylated, neuroprotective state) resulting in stabilization of the neuronal cytoskeleton. sAPP-α is also known to bind SEMA3A and prevent its repulsive effects on the nerve terminal (presumably by preventing SEMA3A binding to NRP1, which leaves CRMP2 in an unphosphorylated, neuroprotective state, thus helping to stabilize the neuronal cytoskeleton). **(C)** Detrimental effects of non-guidance and axon guidance molecules on NMJ stability in ALS. Non-guidance molecules (in purple) such as galectin-1 (gal-1; secreted from TSCs) and amyloid precursor protein (APP; expressed by damaged muscle fibers) can both directly cause synapse degradation (see main text for further detail). Axon guidance molecules (in green) like SEMA3A (expressed by TSCs), NOGO-A (expressed by muscle fibers) and EPHA4 (expressed by motor neurons) are upregulated in ALS mice. SEMA3A and NOGO-A can influence the stability of the neuronal cytoskeleton via activation of the collapsing response mediator protein (CRMP)-pathway. SEMA3A does this via interaction with neuropilin-1 (NRP1) on the plasma membrane of MN, resulting in the phosphorylation of CRMP2. NOGO-A (in muscle) interacts with its receptor, NgR (on MN), to activate the CRMP4-RhoA kinase pathway. CRMP2 and CRMP4 can interact directly with cytoskeletal proteins (microtubulin and actin respectively) to negatively alter their dynamics. EPHA4 (expressed by MN) feeds into the RhoA-kinase pathway, which also regulates cytoskeletal dynamics. **(D)** Potential therapies that target specific molecular components of the NMJ to improve NMJ stability in ALS. The administration of trophic factors (such as VEGF, IGF-1, and GDNF) has been shown to have direct neuroprotective effects and/or effects on TSC survival and maintenance (Krakora et al., 2012). Administration of recombinant gal-1 (gal-1^oxd^; the oxidized form) is neuroprotective; presumably through a mechanism that enhances VEGF-signaling (gal-1^oxd^ binds NRP1, which enhances binding of co-receptor VEGFR2). Administration of β-secretase blockers has the potential to enhance sAPP-α production (by enhancing the non-amyloidogenic cleavage pathway). sAPP-α binds to unphosphorlyated CRMP2 which may help maintain CRMP2 in an non-phosphorylated state and thus aid in the stabilization of the neuronal cytoskeleton. Neutralization strategies using antibodies directed toward NRP1 (Venkova et al., 2014) or NOGO-A (Bros-Facer et al., 2014) have the ability to inhibit these signaling pathways, probably by preventing phosphorylation of the appropriate CRMPs, and thus enhancing neuronal cytoskeleton stabilization. Fasudil, a RhoA-kinase inhibitor, can be administered to prevent EPHA4 function by inhibiting the activation of downstream mediators that usually lead to the destabilization of the neuronal cytoskeleton. The ideal treatment for ALS would therefore combine the various interventions listed above to increase the potential success of the therapeutic approach by enhancing the beneficial pathways and inhibiting the destructive pathways respectively.

As described above mounting evidence suggests that the earliest presymptomatic functional and pathological changes are occurring distally in axons and at the NMJ. These changes precede, and can be independent to, the loss of cell bodies or alterations in other cell types already linked to the ALS disease process (reviewed in Dupuis and Loeffler, 2009). The NMJ is a tri-partite synapse, composed of the skeletal muscle fiber, the motor neuron terminal and several TSCs (see Figure 1A), the latter being important for maintaining synaptic integrity and influencing the plasticity of the NMJ during development and disease (Son and Thompson, 1995; Son et al., 1996; Auld and Robitaille, 2003). Changes which induce pathological features at the NMJ can occur due to intrinsic factors within the motor neuron as discussed above, but recent evidence provides data to show that pathological changes occurring in the skeletal muscle fiber itself and/or in the TSCs can also lead to NMJ destruction prior to motor neuron degeneration (De Winter et al., 2006; Jokic et al., 2006; Dupuis et al., 2009; Gorlewicz et al., 2009).

One physiological aspect that is receiving much attention in terms of an early sign for NMJ destruction is altered synaptic transmission. Recent evidence has demonstrated impaired neuromuscular transmission in presymptomatic (4–6 weeks old) ALS mice compared to age-matched controls (Rocha et al., 2013). Impaired synaptic transmission and other electrophysiological changes in electrical excitability or conduction velocity disrupt the necessary interactions between the nerve terminal, muscle fibers and TSCs required for appropriate postnatal formation of the NMJ, which can lead to a delay in maturation of the NMJ in presymptomatic animals (Blijham et al., 2007; Van Zundert et al., 2008; Caillol et al., 2012). During development, appropriate electrical activity is essential to get the correct distribution of the acetylcholine receptors (AChR) on the muscle cell membrane for NMJ maturation at that location (Goldman et al., 1988), thus one could speculate that the abnormal age-appropriate activity seen at the NMJ of presymptomatic ALS mice could alter the subcellular localization of the AChRs and cause a destabilization of the affected NMJ. In fact, by experimentally maintaining neuromuscular activity in partially denervated muscles vulnerable motor units are able to survive longer over the course of the disease (Gordon et al., 2010), illustrating the importance of appropriate synaptic transmission in the health of the NMJ.

Changes in electrophysiology can be indicative of a disturbance in appropriate cytoskeletal function, and recently an abundance of studies have illustrated the importance of changes in the distal cytoskeleton as a potential trigger for NMJ destruction in ALS. The hallmarks of a distal axonopathy include mitochondrial defects, aggregation of proteins and dysfunctional intracellular transport, and it has been suggested that a disturbance of the distal cytoskeleton is a primary factor causing these hallmark features, and ultimately leads to the retraction of nerve terminals from the NMJ (Soler-Martín et al., 2012). Axonal function heavily depends on a healthy cytoskeleton composed of microfilaments and intermediate filaments (predominantly for structural support) and microtubules (for intracellular transport). Aggregates or abnormalities in neurofilaments (a subclass of intermediate filaments found in neurons) have been directly linked to the ALS phenotype (Williamson et al., 1998; Lalonde and Strazielle, 2003) and pharmacological stabilization of microtubules decreases motor neuron death, and improves life expectancy in ALS mice (Fanara et al., 2007), illustrating the importance of the cytoskeleton in the ALS disease process. Mutations in the profilin gene, an important mediator of actin dynamics, has recently been found to account for a small portion of inherited ALS cases (Wu et al., 2012) further linking defects in the cytoskeleton as a feature of ALS. Additional molecular changes occurring at the NMJ itself, whether originating from the muscle fiber, the TSCs or the motor nerve terminal can be capable of influencing the stability of the synapse during the course of ALS. These will be discussed below, specifically focusing on how inducers of instability or protectors of NMJ integrity are involved in a delicate balance to maintain the appropriate structure and function of the NMJ.

## Molecular mechanisms that govern distal axon and NMJ stability and/or instability

### Neurotrophic factors

Over the last decades, a large body of evidence has supported the administration of neurotrophic factors (such as glial cell line-derived neurotrophic factor [GDNF], insulin-like-growth factor [IGF-1], ciliary neurotrophic factor [CNTF] and vascular endothelial growth factor [VEGF]) to ameliorate the ALS disease process, but with limited success in terms of a clinical use for this approach (Henriques et al., 2010). However, the expression of most of these trophic factors show a remarkable decline over the course of the disease (reviewed in Krakora et al., 2012) suggesting that diminished neurotrophic support is contributing to the disease. Interestingly, specifically targeting the muscle to overexpress these neurotrophic factors (GDNF for example) has led to the most efficient outcomes in terms of improved locomotor performance and increase lifespan, compared to targeting the motor neurons (via the spinal cord) themselves (Li et al., 2007; Suzuki et al., 2008). Both studies reported an increase in the number of NMJs remaining innervated and the number of surviving motor neurons in mid- to late-stages of the disease after intramuscular GDNF administration (Li et al., 2007; Suzuki et al., 2008). Similarly, muscle-mediated IGF expression stabilized neuromuscular junction and enhanced motor neuron survival leading to a delay in disease onset and a slowing of disease progression (Kaspar et al., 2003; Dobrowolny et al., 2005). Intramuscular administration of VEGF has also shown to preserve NMJs and prolong survival (Azzouz et al., 2004; Zheng et al., 2007) and currently holds the most therapeutic potential in terms of a clinical treatment for ameliorating ALS (Keifer et al., 2014). Although CNTF has been shown to have survival promoting abilities for vulnerable motor neurons in ALS mouse models (Pun et al., 2006) and may have a role in compensatory sprouting in motor neuron disease (Simon et al., 2010), due to its lack of positive effect in clinical trials for ALS (adverse effects included an increase in muscle weakness and a higher rate of weight loss in treated individuals; Bongioanni et al., 2004), its suitability as a treatment for ALS definitely requires further assessment.

The mechanisms by which neurotrophic factors modify the axonal cytoskeleton to stabilize the NMJ are not well understood but several recent studies have tried to shed light on this question. During development, the neuronal growth cone directs the developing axon by translating environmental guidance cues into cytoskeletal rearrangements. New evidence has revealed that upon VEGF stimulation, the actin cytoskeleton in the growth cone undergoes immediate and fast rearrangement that enables increased motion (Olbrich et al., 2013). One could speculate that a similar event is occurring at the NMJ in ALS mice upon trophic factor administration; the cytoskeleton of the motor neuron terminal is modified to a highly active state by the presence of the trophic factor at the NMJ enabling it to remain stable in an otherwise destructive environment. Supporting this idea is a recent study whereby CNTF treatment in mice with motor neuron disease was shown to activate a pathway leading to microtubule stabilization, allowing axon elongation and maintenance (Selvaraj et al., 2012).

Interestingly, overexpression of GNDF in muscle, but not in glia, causes hyperinnervation of NMJs, resulting in the formation of multiple end-plates per muscle fiber (Nguyen et al., 1998; Zwick et al., 2001) and leads to earlier regeneration (i.e., increased axonal sprouting) following sciatic nerve crush (Magill et al., 2010). Could muscle-mediated overexpression of neurotrophic factors in a disease or injury paradigm improve the regenerative outcome by also affecting the TSCs present at the NMJ? Peripheral Schwann cells can be categorized into the myelinating variety (positioned along peripheral nerves) and the non-myelinating variety (e.g., the TSCs of the NMJ), their identity being defined by a variety of signals during development (Jessen and Mirsky, 2002). By acting via neurotrophic factor receptors that remain expressed during adulthood in (terminal) Schwann cells (Sondell et al., 1999; Hess et al., 2007; Garcia et al., 2010), muscle-derived overexpression of neurotrophic factors can stimulate (terminal) Schwann cell migration (in the case of GDNF; Iwase et al., 2005) and increase the number of TSCs at the NMJ (in the case of neurotrophin-3, NT-3; Hess et al., 2007). In addition to their maintenance role at the neuromuscular synapse, neurotrophic factors have also been shown to modulate signaling between neurons and glial cells at the NMJ during development (Todd et al., 2007). In this way, a damaged or denervated neuromuscular synapse may benefit from the increased influx of TSCs at and around the synapse (mediated by muscle-mediated overexpression of trophic factors), whose plastic responses in disease or injury models serve to guide nerve sprouting, reinnervation and signaling at the neuromuscular synapse (Son et al., 1996; Tam and Gordon, 2003a; Sugiura and Lin, 2011).

What these studies demonstrate is that maintaining trophic factor expression at the NMJ helps to preserve the NMJ by modifying the cytoskeleton *of the motor neuron*, which translates to a slowing down of disease progression with an improvement in motor performance and life span. However, changes in other molecular pathways are still present and these will also influence the stability of the NMJ. These changes can be roughly divided in two categories: molecules that are involved in various aspects of cell-cell signaling and/or cell adhesion (we refer to these as “non-guidance molecules”) and molecules that are known to govern axon-guidance and/or synapse formation (“guidance molecules”).

### Non-guidance molecules

The cytosolic form of galectin-1 (gal-1) was first linked to the pathophysiology of ALS as a component of axonal neurofilamentous spheroids in sALS and fALS (Kato et al., 2001). Galectins form a large family of proteins involved in mediating cell-cell interactions, cell-matrix adhesion and transmembrane signaling (Camby et al., 2006; Yang et al., 2008). Both gal-1 and gal-3 have been associated with ALS, the latter as a candidate biomarker for the disease (Gonzalez de Aguilar et al., 2008; Zhou et al., 2010). In the terminal stages of ALS (at 120d in the G93A-hSOD1 ALS mouse), an 11-fold increase in gal-3 was identified in motor neurons compared to age-matched non-transgenic controls (Ferraiuolo et al., 2007), but warrants further analysis to understand its role. Gal-1 has been studied in more detail with regards to its role in the disease which seems to be dependent on its localization and oxidative state. A delicate equilibrium exists between monomer and dimer formation, based on the oxidative state of the protein, which in turn gives rise to the various functions of gal-1 (Stowell et al., 2009). Cytosolic, non-oxidized gal-1 is upregulated after injury in all TSCs where it is thought to have a role in the pruning of damaged nerve endings after a lesion (Plachta et al., 2007), but the timing of when gal-1 would be upregulated in ALS is unknown. Based on the work of Plachta et al. (2007) one could expect gal-1 to be first upregulated in the TSCs of FF synapses in ALS as they are the first to shown signs of (morphological) damage during disease progression (Frey et al., 2000), potentially forming part of the molecular signature that gives these synapses their vulnerability to damage. However, this remains to be studied in detail.

*In vitro* evidence supports an early role for gal-1 in distal axonopathy in that processes of cultured wild-type neurons are affected first upon treatment with non-oxidized recombinant gal-1, followed by the cell body (Plachta et al., 2007). Specifically interesting in the context of ALS is the fact that disintegration of the neuromuscular junction is delayed after sciatic nerve lesion in mice lacking the gene for gal-1 (Plachta et al., 2007), supporting the idea that gal-1 has a role in dismantling the NMJ. Remarkably the oxidized, secreted form of gal-1 actually promotes axonal regeneration (Horie and Kadoya, 2004) and when administered to ALS mice in recombinant form, it improves motor activity, delays disease onset and prolongs survival of treated animals (Chang-Hong et al., 2005; Kato et al., 2005), highlighting its potential as a therapeutic strategy to stabilize NMJs. The molecular mechanisms by which the secreted form of galectin-1 promotes axonal regeneration or stabilizes NMJs are still unknown, but a recent study has identified secreted, oxidized gal-1 as a novel ligand for neuropilin-1 (NRP-1), activating the VEGF-signaling pathway to mediate migration and adhesion of endothelial cells (Hsieh et al., 2008). NRP1 is an important component in the VEGF signaling pathway, and in the ALS disease paradigm, treatment with VEGF has been shown to stabilize NMJs, improve motor function and prolong survival in ALS mice (Lambrechts et al., 2003; Azzouz et al., 2004; Storkebaum et al., 2005; Zheng et al., 2007) via its neuroprotective effects on motor neurons (Llado et al., 2013). Thus, one could speculate that a mechanism whereby the VEGF pathway is stimulated via oxidized gal-1 binding with NRP1 on the motor neuron surface to activate the co-receptors necessary for VEGF-signaling, promotes NMJ stabilization with subsequent beneficial effects on the ALS phenotype.

The transmembrane glycoprotein CD44 is a member of the cell-adhesion molecule family of proteins important in extracellular matrix function (reviewed in Goodison et al., 1999), and during development of peripheral nerves it is expressed in glial cells and has a role in maintaining neuron-Schwann cell interactions (Sherman et al., 2000). However, it remains expressed in TSCs at the NMJ (and other non-myelinating Schwann cells) throughout adulthood, but greatly increases in expression in these cells upon disease-related denervation in rat ALS skeletal muscle (Gorlewicz et al., 2009). Interestingly, CD44-positive TSCs are found in NMJs across all types of muscle fibers (Gorlewicz et al., 2009), thus although this molecule influences glial plasticity at the NMJ it is probably not a feature of the selective vulnerability seen at the NMJs of FF motor neurons in ALS (Frey et al., 2000). Instead, the enhanced expression of CD44 (or other cell-adhesion molecules) by TSCs may illustrate a compensatory mechanism to facilitate TSC plasticity in response to pathological denervation-reinnervation, and this could be happening already in the presymptomatic phases of the disease. The study by Gorlewicz and colleagues showed that CD44 was increased in TSCs in end-stage ALS mice compared to age-matched wild-type controls (Gorlewicz et al., 2009) but evidence exists to show that increased expression of other cell-adhesion molecules (such as N-CAM) occurs in parallel with, and may be a compensatory feature against, the early event of fast-twitch muscle denervation (Gordon et al., 2009). During development, N-CAM has an important role in mediating adhesion between neurons, glial cells and muscle cells prior to synaptogenesis (Rutishauser, 1985). Thus, an increase in N-CAM after nerve injury may function to prime denervated skeletal muscle for reinnervation (Covault and Sanes, 1985). In the context of ALS, one can speculate that an increased expression of N-CAM (or other cell adhesion molecules, such as CD44) may be a compensatory reaction to the denervation that is occurring in an effort to maintain the muscle fiber in a state suitable for reinnervation.

Recently, an increase in colocalization between CD44 and ErbB3 in the TSCs of ALS rodents was identified (Gorlewicz et al., 2009). Members of the ErbB family are important receptors in the neuregulin (NRG1) pathway which mediates neuromuscular synapse formation by maintaining the postsynaptic specialization in the muscle and thereby enhances synaptic transmission at the NMJ (Trachtenberg and Thompson, 1997; Schmidt et al., 2011). In addition, ErbB expression and the NRG1 pathway in (terminal) Schwann cells is necessary for the proliferation, migration and survival of these cells during development (Riethmacher et al., 1997; Garratt et al., 2000; Wolpowitz et al., 2000). Given that TSCs play an important role in NMJ plasticity and axonal sprouting after injury and in disease paradigms (Tam and Gordon, 2003a,b; Gordon et al., 2004), one can assume that by concentrating ErbB receptors on the membrane of the TSCs as mediated by CD44 expression (Gorlewicz et al., 2009), the NRG1-ErbB pathway is enhanced within TSCs and may boost their protective abilities at the NMJ by potentially controlling TSC-derived neurotrophin production (Garratt et al., 2000). Interestingly, mutations in the ErbB4 gene leading to disruption of the NRG1-ErbB pathway has been shown to cause a clinical subset of ALS (Takahashi et al., 2013), supporting the importance of this pathway as a protective mechanism against the disease.

Additionally, CD44 has also been found to be upregulated in astrocytes and microglia in the spinal cord of 12 week old ALS mice, the time at which the clinical phase of the disease begins, and increases over time (Matsumoto et al., 2012). In injured spinal cord, chondroitin sulfate proteoglycans in the extracellular matrix can act via CD44 to promote the production of IGF-1 by astrocytes and microglial cells (Rolls et al., 2008). Could the extracellular matrix at the NMJ in muscle be using CD44 in TSCs to stimulate IGF-1 production, a trophic factor which, when exogenously administered, is known to promote NMJ stability and improve the ALS phenotype (Kaspar et al., 2003; Dobrowolny et al., 2005)?

The amyloid precursor protein (APP), a membrane protein with the ability to regulate synapse formation and repair (Priller et al., 2006; Nalivaeva and Turner, 2013; Octave et al., 2013) has been widely studied for its role in Alzheimer's disease, but its role in other neurodegenerative diseases such as ALS continue to be discovered (Muresan et al., 2014). During (post-natal) development of the NMJ, APP expressed by the skeletal muscle is involved polyneural synapse elimination to create a mono-innervated synapse through interaction with post-synaptic components involved in junction formation (Akaaboune et al., 2000; Choi et al., 2013). The extracellular fragment of APP has been shown to act via the Death Receptor 6 (DR6) apoptotic pathway to regulate axonal behavior during development, and the activation of this pathway due to loss of trophic support has been linked to peripheral nerve degeneration after axonal damage in adulthood (Nikolaev et al., 2009). Following post-natal maturation of the NMJs, APP is downregulated in adult skeletal muscle (Akaaboune et al., 2000). However, APP is found to be elevated in muscle prior to the appearance of clinical symptoms in both ALS mice and in human patients (Koistinen et al., 2006) and in motor neurons in presymptomatic ALS mice (Ferraiuolo et al., 2007). Its role in ALS was further defined by Bryson and colleagues who showed that APP expression at the muscle is triggered by denervation and, notably, is specific to Type IIb fibers (Bryson et al., 2012), these fibers being the first to become denervated in ALS (Frey et al., 2000). In addition, genetic ablation of APP improves innervation, motor function and motor neuron survival in ALS mice, further emphasizing its importance in the pathophysiology of ALS (Bryson et al., 2012). Importantly, removal of APP did not extend survival in the ALS mice, suggesting that the detrimental effects of APP contribute to the onset and early phases of the disease (Bryson et al., 2012). Given the fact that a lack of APP can enhance the number of functional synapses *in vitro* (Priller et al., 2006), presymptomatic expression of APP (in the muscle or the neuron) may initiate synaptic apoptosis at the NMJ in ALS (perhaps due to reactivation of its role in the elimination of multi-innervated synapse during development; (Akaaboune et al., 2000). In conjunction with other destructive factors (discussed below), normal synaptic function is compromised, which leads to further denervation of the synapse and the progressive neuronal degeneration typical of the disease.

### Guidance molecules

Various axon guidance cues, with important roles during neuronal development, are now being linked to the pathophysiology of ALS, and more importantly, to the presymptomatic stages of the disease (Schmidt et al., 2009). These include semaphorin3A, EphrinA4 and NOGO-A. The secreted repulsive axon guidance cue semaphorin3A (SEMA3A), is selectively upregulated in TSCs in presymptomatic ALS mice, specifically at the NMJs on Type IIb muscle fiber (De Winter et al., 2006). Further evidence implicates the SEMA3A signaling pathway in ALS. First, ALS-mice with motor neuron specific knockout of the receptor for SEMA3A, neuropilin-1 (NRP1), display improved motor function (Moloney et al., 2012). Second, treatment of ALS-mice with a monoclonal antibody that interferes with SEMA3A-NRP1 signaling prolonged survival and improved motor function (Venkova et al., 2014). These improvements are thought to be due to the disruption of signaling to the downstream effectors of the SEMA3A signaling pathway, the collapsin response mediator proteins (CRMPs), which are known to mediate various roles during development and maturation, including neuronal migration, synapse formation, and synaptic plasticity by switching their phosphorylation status (Yamashita and Goshima, 2012). Upon SEMA3A binding to NRP1, CRMPs are phosphorylated and this facilitates SEMA3A-mediated growth cone collapse by causing the dismantling of cytoskeletal structures *in vitro* (Goshima et al., 1995; Deo et al., 2004; Schmidt and Strittmatter, 2007; Hida et al., 2012). In presymptomatic ALS mice, CRMP4a is upregulated in a subset of lumbar motor neurons, and was shown to be directly linked to motor neuron degeneration (Duplan et al., 2010), presumably through its ability to modulate the actin cytoskeleton (Alabed et al., 2007). Specific population-associated mutations within the CRMP4a gene have recently been found to cause a shortening of survival in affected motor neurons by impairing axonal growth (Blasco et al., 2013). With this in mind it is plausible that the subpopulation of motor neurons that express (mutant) CRMP4a correspond to the susceptible FF motor neurons synapsing on Type IIb/x fibers, but this has yet to be confirmed.

Interestingly, although intramuscular AAV-mediated overexpression of CRMP4a leads to a significant increase in denervation and motor neuron death in WT mice, there were no such effects after CRMP2 overexpression (Duplan et al., 2010), which may point to differences in the way each CRMP exerts its effects on the cytoskeleton. The effects of CRMP2 on the cytoskeleton seem to be regulated by several mechanisms, most importantly by the phosphorylation state of CRMP2 itself which leads to disruption of microtubule assembly (Gu and Ihara, 2000; Yoshimura et al., 2005; Yamashita and Goshima, 2012). As phosphorylation of CRMP2 is important in mediating CRMP2's negative effects on the cytoskeleton and is facilitated by SEMA3A binding (Deo et al., 2004; Uchida et al., 2005; Hensley et al., 2011; Khanna et al., 2012), overexpression of CRMP2 alone, especially in an uninjured system where SEMA3A is not present, may not result in adequate phosphorylation of CRMP2 to allow for cytoskeletal abnormalities and subsequent motor neuron degeneration as mediated by CRMP4a overexpression (Duplan et al., 2010). In addition, abnormal phosphorylation of CRMP2 has been identified as a characteristic feature of Alzheimer's disease (Williamson et al., 2011) and further work is focusing on its role in other neurodegenerative disorders in terms of cytoskeletal alterations (Hensley et al., 2011). In its unphosphorylated state, CRMP2 can actively stimulate neurite outgrowth (Yoshimura et al., 2005) thus by inhibiting SEMA3A signaling one could promote the accumulation of unphosphorylated CRMP2 in an effort to create an environment at the NMJ that is more permissible to maintaining the synapse in a healthy state. Studies focusing on the (pharmacological) stabilization of CRMP2 in its unphosphorylated state are also promising for their potential as treatments for ALS as well as other neurodegenerative disorders (Hensley et al., 2010, 2011; Khanna et al., 2012).

In addition, protein-protein interactions that modulate CRMP2 function have been identified. Targeted disruption of retrograde transport is known to result in neuromuscular changes consistent with progressive motor neuron diseases (LaMonte et al., 2002; Puls et al., 2003) and SOD1-mutant ALS mice display impaired axonal transport months prior to disease onset which could indicate that decreased transport is correlated with development of motor neuron disease (Williamson and Cleveland, 1999). Active transport along microtubules requires kinesin and dynein and CRMP2 has been found to bind dynein and negatively regulate dynein-mediated retrograde transport (Arimura et al., 2009; Rahajeng et al., 2010). In this scenario it seems that CRMP2 acts as a linking protein between the molecular motors and the microtubule cytoskeleton, but it is not clear from these studies whether this function is dependent on CRMP2's phosphorylation state. CRMP2 function can also be directly modulated by other members of the CRMP family. Unphosphorylated CRMP2 promotes axonal outgrowth by assisting tubulin assembly (Fukata et al., 2002; Yoshimura et al., 2005), but this function can be inhibited by the presence of CRMP5 (Brot et al., 2010) providing another potential point at which CRMP2 function can be manipulated by targeted therapeutic approaches against CRMP5 function.

Additionally, CRMP2 has been found to be a putative binding partner for sAPP-α (Pawlik et al., 2007), a soluble, secreted ectodomain isoform produced by α- and γ-secretase cleavage of the main neuronal APP through the non-amyloidogenic pathway. In the amyloidogenic pathway APP is sequentially cleaved by β-secretase followed by γ-secretase to produce the amyloid-beta product, well known for its involvement in neuronal degeneration in Alzheimer's disease (O'Brien and Wong, 2011). Interestingly, sAPP-α is neuroprotective, enhances neurite elongation and modulates synaptic plasticity (Turner et al., 2003), so perhaps the interaction between sAPP-α and CRMP2 strengthens their neuroprotective effects. Further supporting this idea is the finding that sAPP-α itself can directly interact with SEMA3A and prevent SEMA3A-induced growth cone collapse *in vitro* (Magdesian et al., 2011). Therapeutic approaches that would enhance the cleavage of APP to sAPP-α (e.g., by inhibiting β-secretase function) could effectively “kill two birds with one stone”: APP bioavailability decreases and the SEMA3A signaling pathway is inhibited by the presence of sAPP-α. In this way modulating β-secretase function and APP bioavailability could potentially have a positive effect on the stability of the cytoskeleton at the NMJ and has already been shown to delay the onset of neuronal degeneration in ALS (Rabinovich-Toidman et al., 2012). However, one has to proceed with caution as β-secretase is required for NRG1-mediated myelination of the peripheral nerve (Hu et al., 2006; Willem et al., 2006) and therefore inhibition may alter the beneficial effects of NRG1 signaling in Schwann cells (see above for details regarding the NRG1 pathway in ALS). One way around this may be to enhance the dimerization of APP itself with small molecule compounds, which alters APP conformation and influences the ratio of cleavage by α- and β-secretase to result in significantly higher levels of sAPP-α (Libeu et al., 2012).

SEMA3A expression may also be a part of the myogenic program necessary for muscle regeneration after muscle injury. It has not yet been studied in the context of ALS, but given that satellite cells become activated after muscle injury or denervation (Seale and Rudnicki, 2000) and begin to express and secrete SEMA3A (Sato et al., 2013) it is possible that, upon disease-related denervation, satellite cells in an ALS muscle would also begin to produce SEMA3A. An array of growth factors modulate the expression of SEMA3A (Tatsumi et al., 2009; Do et al., 2011) giving rise to the idea that SEMA3A, expressed by satellite cells, may serve a beneficial role in terms of skeletal muscle regeneration in that it delays neuronal sprouting and re-attachment of nerve terminals until damaged muscle fibers have been restored. The myogenic pathway is active in presymptomatic ALS mice, but as the disease progresses, the function of satellite cells becomes impaired (Pradat et al., 2011; Manzano et al., 2013) and levels of myogenic proteins decrease even though RNA levels remain high (Manzano et al., 2011). If the myogenic process is unable to maintain muscle regeneration, muscle fibers are not restored and SEMA3A continues in its role in delaying re-attachment of terminals. This, in combination with other destructive factors occurring at the NMJ, could result in a “snowball” effect of NMJ degeneration.

EphrinA4 (EPHA4), a receptor tyrosine kinase, is a member of the ephrin family known to have well-established functions in axonal repulsion during neuronal development. During adulthood EPHA4 can regulate synapse formation and plasticity (Klein, 2009). In human ALS patients, low levels of EPHA4 have been associated with later disease onset and/or a slower disease progression, providing a site for potential therapeutic intervention (Van Hoecke et al., 2012). Furthermore, large motor neurons, which are more vulnerable to degeneration, were found to express higher quantities of EPHA4 mRNA in presymptomatic ALS providing additional evidence for the differential vulnerability of motor neurons in ALS (Van Hoecke et al., 2012). Inhibition of EPHA4 genetic ablation, was also shown to rescue motor neuron degeneration in ALS mice and increase the number of NMJs being re-innervated after sciatic nerve axotomy (Van Hoecke et al., 2012). These data point to EPHA4 being a vital member of the molecular repertoire that causes a motor neuron to be more susceptible to degeneration. Interestingly, inhibition of EPHA4 also rescues other motor neuron deficits in TDP-43 or SMN1-induced axonopathies which may indicate that the EPHA4 is independent to how degeneration occurs but instead maybe be a “generic determinant of vulnerability of neurons to degeneration” (Van Hoecke et al., 2012). Pharmacological studies have identified the potential of fasudil (an inhibitor of rho-kinase function, a downstream mediator of EphrinA signaling) for improving motor function after stroke (Lemmens et al., 2013) and limits motor neuron loss in ALS mice (Van Hoecke et al., 2012; Takata et al., 2013). As a results, a Phase II open-label trial (ClinicalTrial.gov: NCT01935518) was initiated in September 2013 to identify the efficacy of fasudil treatment in slowing functional decline in ALS patients, with results expected in 2015.

To date, NOGO-A has been the most promising in terms of therapeutic potential in relation to NMJ destruction in ALS pathophysiology. This myelin-derived protein is known to act on the cytoskeleton to exert inhibitory effects on neurite outgrowth, and is a major factor in the failure of CNS axons to regrow after injury (Pernet and Schwab, 2012). *In vitro* evidence shows that stimulation of neuronal growth cones with NOGO-A results in colocalization of CRMP4 and RhoA to mediate actin-dependent cytoskeletal rearrangements (Alabed et al., 2007). NOGO-A is highly expressed in skeletal muscle of presymptomatic ALS rodents and in clinical defined ALS patients and may even be a prognostic marker, its levels correlating with the severity of motor impairment as the disease progresses (Dupuis et al., 2002; Jokic et al., 2005; Pradat et al., 2007). A clear link to NMJ integrity was illustrated whereby genetic ablation of NOGO-A in ALS mice reduced muscle denervation and increased survival, and overexpression of NOGO-A in muscle of wild-type mice destabilized NMJs (Jokic et al., 2006). NMJ destabilization by NOGO-A is consistent with the fact that NOGO-A signaling has been linked to synaptic plasticity and stability in hippocampal neurons (Peng et al., 2011) and in the adult cerebral cortex (Akbik et al., 2013). A recent study has illustrated the potential of anti-NOGO-A antibodies in significantly improving neuromuscular function even when administered to ALS mice in the symptomatic stages of the disease (Bros-Facer et al., 2014). Currently a Phase II trial is underway using anti-NOGO-A antibodies (GSK; Ozanezumab) in an effort to neutralize NOGO-A function in ALS patients (ClinicalTrials.gov: NCT01753076), with primary results due in May 2015.

## Conclusion

Ultimately, ALS is a multifaceted disease with multiple modifiers that enhance axon retraction and/or destabilize the NMJ and therefore a more effective approach to designing therapeutic interventions is to consider all (known) components that influence NMJ integrity (see Table 1 for an overview of molecules discussed in this review). The interventions discussed above (enhanced expression of neurotrophic factors, neutralization of inhibitory axon guidance cues) individually do not prevent the disease. Although disease onset is delayed by certain interventions in the neurotrophic factor, “non-guidance” or “guidance” molecule pathways, resulting in increased motor neuron survival and motor performance, the disease phenotype ultimately manifests itself. Intrinsic pathways, including those mediated by cell-adhesion molecules, are present to compensate for denervation but ultimately fail to overcome the rate and level of denervation (see Figure 1B). Combination therapies which aim to inhibit the destructive features and enhance the compensatory features could be a powerful way to halt the disease process in the presymptomatic stage (see Figure 1D).

**Table 1:**
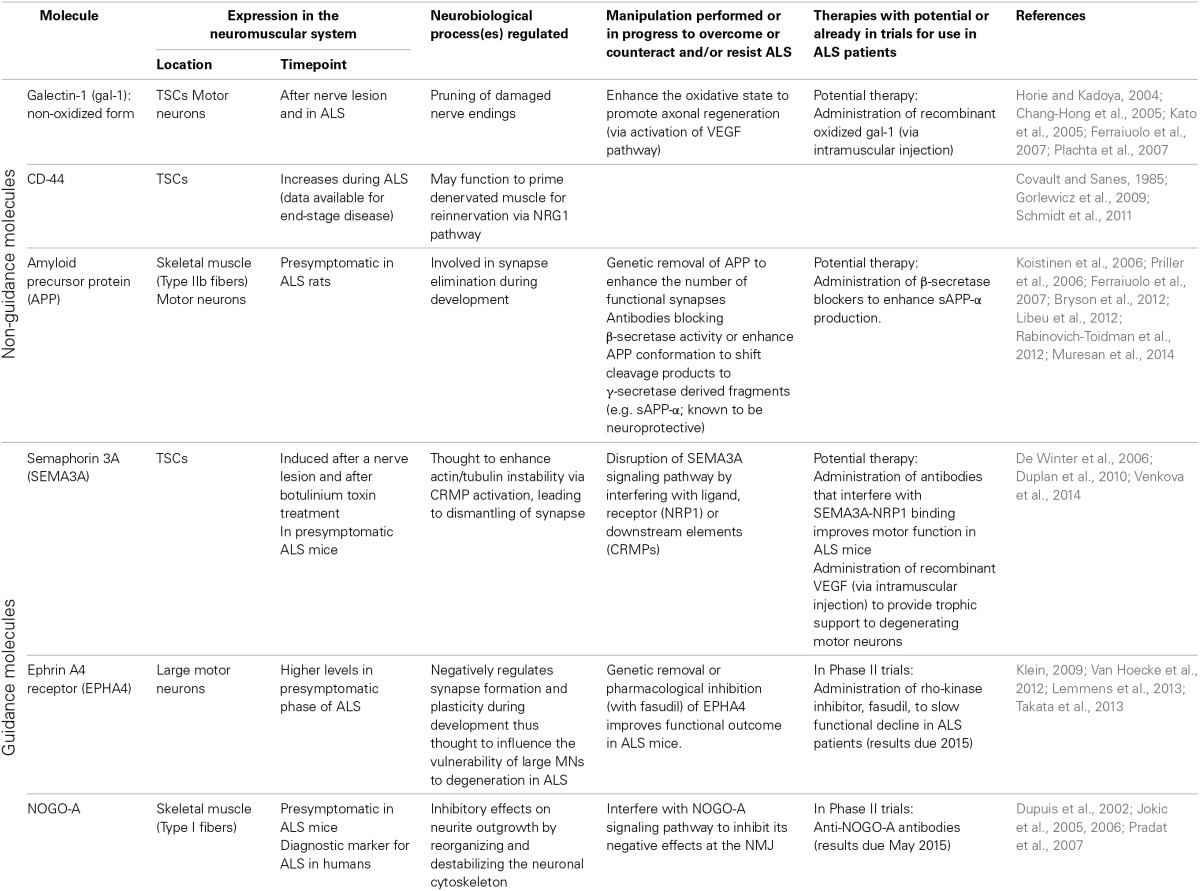
**Overview of non-guidance and axon guidance molecules expressed at the neuromuscular junction and implicated in distal motor axon degeneration in ALS**.

As discussed, NMJ destruction may begin due to a combination of factors; vulnerable neurons display altered cytoskeletal properties due to the effects of external guidance cues present in the vicinity of the synapse (see Figure 1C). By combining several therapeutic approaches that interfere with the effects of guidance molecules specifically, one could create an environment that allows for the NMJ to remain stable in the context of ALS (see Figure 1D). By inhibiting EPHA4, the vulnerability of neurons to degeneration could be reduced thus creating more resistant motor neurons; by inhibiting SEMA3A secreted by TSCs, the TSCs could continue to maintain the NMJ in an suitable state, allowing appropriate neurotransmission to occur between the motor neuron and muscle fibers whereby Type IIb fibers could remain functional for longer; by inhibiting NOGO-A, the Type I fibers could remain functional for a longer period decreasing the severity of (any) motor dysfunction. By approaching the therapeutic intervention from the three sides of the NMJ (the motor neuron, the muscle fiber and the TSCs), the NMJ may perhaps be stimulated to remain in a healthy state for longer, and therefore the onset of the disease might be more efficiently postponed than with individual interventions. By combining this approach with therapies like application of neurotrophic factors, one hopes to enhance pathways that are already known to improve NMJ plasticity and stability, and thus strengthen the potential benefit of the therapeutic approach.

### Conflict of interest statement

The authors declare that the research was conducted in the absence of any commercial or financial relationships that could be construed as a potential conflict of interest.
